# Application of Targeted Next-Generation Sequencing for the Investigation of Thalassemia in a Developing Country: A Single Center Experience

**DOI:** 10.3390/diagnostics13081379

**Published:** 2023-04-10

**Authors:** Razan Hayati Zulkeflee, Rosnah Bahar, Marne Abdullah, Muhammad Amiro Rasheeq Mohd Radzi, Alina Md Fauzi, Rosline Hassan

**Affiliations:** 1 Hospital Universiti Sains Malaysia, Universiti Sains Malaysia, Kubang Kerian 16150, Malaysia; 2 Department of Hematology, School of Medical Sciences, Universiti Sains Malaysia (USM), Kubang Kerian 16150, Malaysia; 3 Department of Paediatrics, School of Medical Sciences, Health Campus, Universiti Sains Malaysia, Kubang Kerian 16150, Malaysia; 4 Faculty of Medicine and Health Sciences, Universiti Sains Islam Malaysia, Bandar Baru Nilai, Nilai 71800, Malaysia

**Keywords:** thalassemia, targeted next-generation sequencing, developing country

## Abstract

Thalassemia is identified as a prevalent disease in Malaysia, known to be one of the developing countries. Fourteen patients with confirmed cases of thalassemia were recruited from the Hematology Laboratory. The molecular genotypes of these patients were tested using the multiplex-ARMS and GAP-PCR methods. The samples were repeatedly investigated using the Devyser Thalassemia kit (Devyser, Sweden), a targeted NGS panel targeting the coding regions of hemoglobin genes, namely the HBA1, HBA2, and HBB genes, which were used in this study. There were many different genetic variants found in 14 unrelated cases. Out of all fourteen cases, NGS was able to determine an additional -50 G>A (HBB:c.-100G>A) that were not identified by the multiplex-ARMS method, including HBA2 mutations, namely CD 79 (HBA2:c.239C>G). Other than that, CD 142 (HBA2:c.427T>C) and another non-deletional alpha thalassemia and alpha triplication were also not picked up by the GAP-PCR methods. We illustrated a broad, targeted NGS-based test that proposes benefits rather than using traditional screening or basic molecular methods. The results of this study should be heeded, as this is the first report on the practicality of targeted NGS concerning the biological and phenotypic features of thalassemia, especially in a developing population. Discovering rare pathogenic thalassemia variants and additional secondary modifiers may facilitate precise diagnosis and better disease prevention.

## 1. Introduction

Hemoglobinopathies are classified as thalassemia, due to the reduced synthesis rate of one of the globin chains or structural hemoglobin (Hb) variants caused by single amino acid substitutions in the α or β globin chains. Thalassemia is an autosomal recessive disorder. Most conditions are found throughout the Middle East, Mediterranean region, and Indian subcontinent, as well as in Southeast Asia [1]. Thalassemia is identified as a prevalent disease in Malaysia, known to be common in the developing country. It is estimated that around 6.8% of Malaysians are thalassemia carriers, with various degrees of anemia [2,3].

There are 8023 Thalassemia patients reported in the National Thalassemia registry as of May 2019, and 5448 (~70%) of them are transfusion-dependent. The probability of surviving up to the age of 60 increased from 60% in 1999 to 80% in 2013 with the advancement in patient diagnosis and management care. The carrier rate was estimated to be every 1 in 20 Malaysian (6.8%). Since 2017, the Malaysia Health Ministry has implemented a screening program among 16-year-old school-age children as part of a prevention and control program. It was found that 9.8% of students were carriers of thalassemia and hemoglobinopathy [3,4].

The impact of reduced hemoglobin formation forms a fragile and weak erythrocyte and leads to chronic hemolytic anemia; therefore, the affected babies will progressively become severely anemic, requiring life-long blood transfusions [5]. Thus, to improve the survival rates and continuation of normal growth in these patients, the treatment is usually accompanied by iron chelation therapy, following regular blood transfusion, to reduce iron overload [6].

The genetic basis of hemoglobin consists of amino acids with a balanced pairing of α-like and β-like globin dimers, which form functional structures and tetrameric units. The α-globin gene cluster comprises three functional globin genes, the embryonic ζ gene (HBZ) and two fetal/adult α (α1 and α2) genes (HBA1 and HBA2), which are located on the short arm of chromosome 16. On the other hand, β-like globin chains on the short arm of chromosome 11 contain five functional genes, the embryonic ε gene (HBE), two fetal Gγ and Aγ genes (HBG2 and HBG1), and adult δ and β (HBD and HBB) genes [7].

Thalassemia is caused by a broad spectrum of point mutations or/and gene deletions, resulting in the reduced or zero formation of alpha or beta globin chain sub-units [3]. The three most common β-globin mutations seen among Malays (73.1%) with the β + thalassemia phenotype are HbE [CD 26 (CAG→AAG)], IVS 1-5 (G→C), and IVS1-1 (G→T). On the other hand, five common β-globin mutations among Chinese (90%) in Malaysia are CD 41/42 (-TCTT), IVS2-654 (C→T) (β + thalassemia phenotype), -28(A→G) (β + thalassemia phenotype), CD 17 (A→T), and CD71/72 (+A) [8]. For α-thalassemia, the most common deletional and non-deletional mutations were --SEA, -α3.7, and -α4.2 and ααCd59, ααCS, and Hb Quong Sze (αα125, respectively [9]). Table 1 summarizes the incidence rate of common molecular characteristics of alpha and beta-thalassemia in several developing countries.

The current investigation for thalassemia requires full blood count, HB analysis, and DNA analysis for a definitive diagnosis. However, there are many limitations in the current diagnosis approach, leading to misdiagnosis in cases with normal or borderline red blood cell indices, normal HbA2 levels, or complexity of the disease due to gene interactions.

The evolution of clinical molecular testing in the genomics era allowed next-generation sequencing analysis to play a significant role in definitive diagnosis-making. Hence, this study was performed to review the application of targeted next-generation sequencing as part of the investigation of thalassemia in a country with limited resources.

## 2. Materials and Methods

A total of fourteen cases diagnosed with thalassemia were recruited from Hematology Laboratory, Universiti Sains Malaysia, Malaysia. These samples tested positive for thalassemia during the screening tests, which tested for red cell indices and peripheral blood film and performed hemoglobin analyses.

Full blood counts (hemoglobin (Hb), mean corpuscular volume (MCV), mean corpuscular hemoglobin (MCH), and red blood cell (RBC)) were tested using the Automated Hematology Analyzer Sysmex XN-1000™ (Sysmex Corporation, Kobe, Japan). Hb analysis was performed using high-performance liquid chromatography (HPLC) (Bio-rad Variant II System, Beta-thalassemia Short Program, Bio-Rad Laboratories, Hercules, CA, USA) to quantify hemoglobin subtypes as HbA2 and HbF. Positive thalassemia screening was defined as having an MCV or MCH less than 80 fl or 27 pg, respectively, and an increase in HbA_2_ of more than 4%.

Positive screening was then followed by genotype results obtained using five different multiplex amplification refractory mutation system (MARMS)-PCRs and one single ARMS-PCR reaction for beta-thalassemia and a gap-polymerase chain reaction (GAP–PCR) for alpha-thalassemia. MARMS were designed for 20 types of β-gene mutations. The mutations identified were MARMS-A (CD 41/42 (-TTCT)), IVS 1-5 (G>C), CD 26 (G>A), CD 17 (A>T), MARMS-B (CD 71/72 (+A)), IVS 1-1 (G>T), CD 8/9 (+G), -28 (A>G), MARMS-C (CD43 (G>T)), Poly A (A>G), IVS1-1 (G>A), CD 16 (-C), MARMS-D (-88 (C>T)), and Cd 15 (G>A) 203), MARMS-E (-86 (C>G)), and CAP + 1 (A>C); Codon 19 (A>G) and ARMS F consisted of IVS 2-654 only. After that, zygosity testing was performed to classify them as homozygous or heterozygous. In contrast, GAP-PCR identified only *α*^3.7^ and *α*^4.2^ types, -^SEA^ deletion, -^THAI^ deletion, and -^FIL^ deletion. In addition, clinical and laboratory data were retrieved and tabulated in Table 2. This study was approved by the Universiti Sains Malaysia Research Ethics Committee JEPeM Code: USM/JEPeM/14120494) and the National Medical Research Register, Medical Research Ethics Committee (MREC), Amendment Approval number NMRR-12-980-13829 (IIIR), and carried out following the Declaration of Helsinki.

Patients’ DNA samples were retrieved, and the multiplex PCR full method was used to amplify the entire *HBA1*, *HBA2*, and *HBB* genes. Targeted NGS for thalassemia libraries were constructed using the Devyser Thalassemia NGS assay (Devyser, Hägersten, Sweden) following the manufacturer’s instructions. This targeted NGS assay specifically identified sequence variants in HBA1, HBA2, and HBB and common sequence variants, including exon spanning copy number variations (CNVs), small or large insertion and deletions (indels), and single-nucleotide variants (SNVs). DNA samples were sequenced using MISeq (NGS platform). A specialized bioinformatic software (Devyser Amplicon Suit v3.5) workflow was used for data analysis, especially for identifying specific deletion parts of the HBA1, HBA2, and HBB genes and for accurate CNV identification. All of the data were saved in the cloud.

## 3. Results

The genetic variants in globin genes were found in 14 unrelated cases, as shown in Table 2. Based on the screening test, four were identified as heterozygous alpha-thalassemia, with two heterozygous HbE and beta-thalassemia compounds, one HbE trait with the coinheritance Hb Constant Spring, and one compound heterozygous beta-thalassemia, and six beta-thalassemia were identified. As MARMS- and GAP-PCR are two different types of methods, they required different requests, and a few of the cases missed sending the sample for either method.

Case 1 was a three-year-old girl with severe hypochromic microcytic anemia. Variants HBB:c.79G>A and HBB:c.92 + 1G>T were found in the patients. HBB:c.79G>A signifies HbE thalassemia, forming an abnormal βE-globin chain [17]. HBB:c.92 + 1G>T (IVS-I-1) is β^0^-thalassemia [18]. Therefore, this patient had compound heterozygous HbE/β^0^-thalassemia, which explained the severity of the anemia.

Cases 3, 4, 5, and 6 were confirmed to have α-thalassemia of deletional types, which were α 4.2 deletions, SEA-type deletions, THAI-type deletions, and α 3.7 deletions, respectively. In Case 3, CD26 (HBB:c.79G>A) and αα/-α^4.2^ were detected by NGS detect. Nevertheless, all cases, including 3, 4, 5, and 6, were asymptomatic.

**Figure 1:**
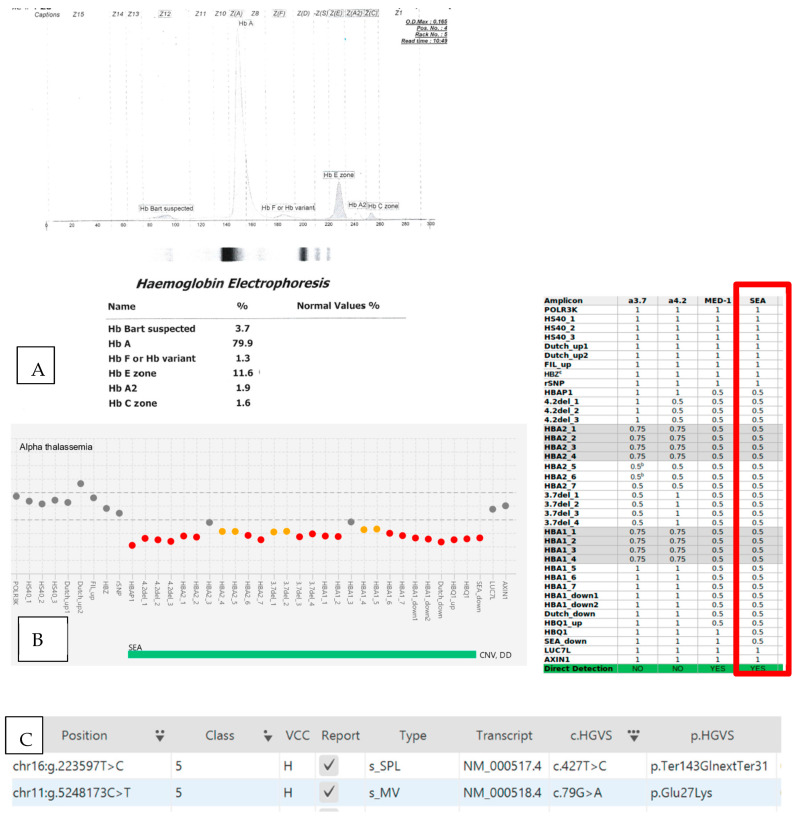
(**A**) Capillary electrophoresis of Case 7, which showed the presence of 1.6% HbCS at Zone 2, 11.6% HbE at Zone 4, and Hb Bart’s (3.7%) at Zone 12. (**B**,**C**) explains that αα/--SEA for alpha-thalassemia and CD 26 (HBB:c.79G>A) and CD 142 (HBA2:c.427T>C) for beta thalassemia were identified simultaneously from the DEVYSER Software. The table on the right side is the expected coverage in *CNV* analysis for amplicons in the *HBA* gene complex. (1 indicates no *CNV*, while 0.5 indicates deletion, and 0 indicate absence of coverage) (Red dot indicates *CNV* less than or equal to 0.60 or greater than or equal to 1.66, while orange dot indicates, *CNV* between 0.60 and 0.7 and between 1.50 and 1.66 respectively) (^b^ These amplicons may not be affected by the several types of 3.7-deletion and triplication, leading to an absence of CNV, ^c^ “HBZ” Amplicons may demonstrate benign deletions or duplications or polymorphisms.)

Case 7 was a 27-year-old female diagnosed with compound heterozygous HbE, with Hb Constant Spring identified (Figure 1). Her hemoglobin was 7.5 g/dL, with a normal RBC of 4.98 and hypochromic microcytic indices. Based on the HPLC method, her HBA, HBA2/E, and HbF were 81.9%, 14.6%, and 3.5%, respectively. On the following date, alpha-deletional and beta-molecular studies were performed on the patient, identifying SEA deletion and CD 26 (*HBB*:c.79G>A). No non-deletional molecular analysis was available at this center to confirm Hb Constant Spring. Her sample was sent for NGS and variant SEA deletion, and HBB:c.79G>A and CD 142 (HBA2:c.427T>C) were identified. CD 142 (HBA2:c.427T>C), known as HB Constant Spring, is a labile α-globin variant causing α-thalassemia, due to a common missense mutation of the termination codon of *HBA2*; Term→Gln [19].

**Figure 2:**
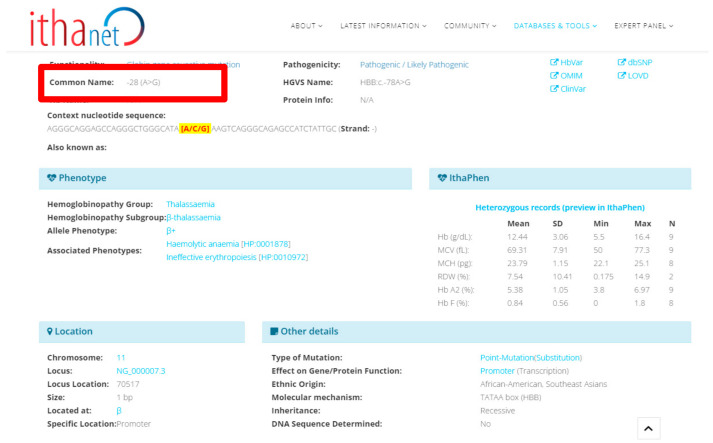
To use an example, in case 9, the sequencing position was confirmed by the ITHANET Portal (one of the databases of variations involving thalassemia or hemoglobinopathies).

Cases 8 and 9 were β-thalassemia patients with IVS I-5 (HBB:c.92 + 5G>C) and -28 (A>G) (HBB:c.-78A>G), respectively (Figure 2). All the cases had a mild degree of anemia with hypochromic microcytic. Both were β + mutations and common beta-thalassemia variants found among Asian populations [20].

Interestingly, case 10 was an 18-year-old girl with severe hypochromic microcytic anemia. Her HbA2 and HbF were 4.3% and 5.4%, respectively. Based on her M-ARMS, heterozygous CD 41/42 was identified. Using NGS, CD 79 (HBA2:c.239C>G), which is known as hemoglobin [Hb] J-Singapore, was found in addition to CD 41/42 (HBB:c.126_129del).

Similarly, case 11 was a 7-year-old girl with mild anemia. HPLC exhibited A2/E and HbF, which were 42.6% and 2.2% respectively. Her molecular genetic by MARMS-PCR showed the presence of CD 26 (G>A) and a CAP + 1 (A>C) allele mutation. Using NGS analysis, her results exhibited as CD 26 (HBB:c.79G>A) and CAP + 1 (HBB:c.-50A>C), with an additional CD 142 (HBA2:c.427T>C), which is the Hb Constant Spring. CAP + 1 (A>C) is one of the rare, silent β-thalassemia formerly found in Asian Indians. Patients with these compound heterozygotes or CAP + 1 mutations normally exhibit borderline hemoglobin (Hb) levels, mean corpuscular volumes (MCV), and Hb A2 levels [21]. In this case, the Hb Constant Spring was missed by HPLC, while CE was not available at that time.

**Figure 3:**
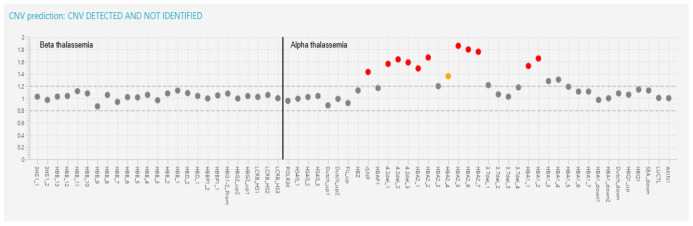

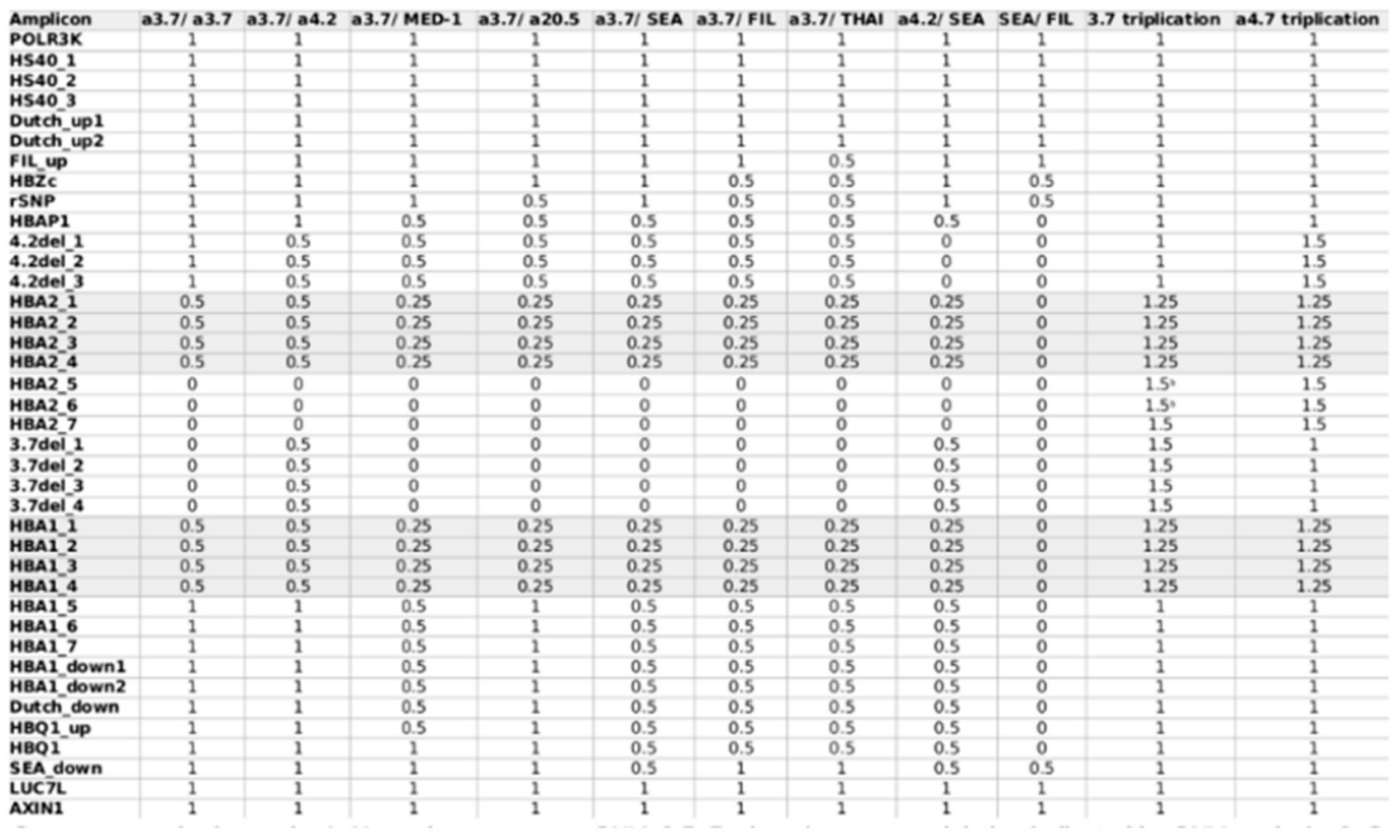
Case 12 was a case of 4.2 triplication detected by the DEVYSER Software. The table below is the expected coverage in the CNV analysis for amplicons involving HBA gene complex duplications/triplications. A 1.5 was indicative of duplication indicated by CNV analysis. (Red dot indicates *CNV* less than or equal to 0.60 or greater than or equal to 1.66, while orange dot indicates, *CNV* between 0.60 and 0.7 and between 1.50 and 1.66 respectively).

Case 12 was a 35-year-old lady with severe hypochromic microcytic who was on regularly packed cell transfusion, despite having heterozygous CD 8/9 (+G), based on her MARMS-PCR. Interestingly, additional triplicated α-globin genes were identified following targeted NGS, which explained the manifestation of her disease (Figure 3).

Case 13 was a case of Hb Malay, confirmed by both MARMS and NGS. The patient had normal hemoglobin with hypochromic microcytic RBC indices. His Hb analysis revealed a high A2 at 4.7% and a slightly raised HbF at 1.4%.

Lastly, case 14 was a 5-month-old boy with severe anemia. He had hypochromic microcytic RBC indices. His Hb analysis showed high a HbF at 95.7%, and interestingly, HbA2 was within range at 2.4%. His M-ARSMs and NGS showed compound heterozygous CD17 (A>T) and IVS II-654 (C>T).

## 4. Discussion

Based on the Thalassemia International Federation Guidelines for the Management of Transfusion-dependent Thalassemia (2021), the thalassemia diagnosis needs to be initiated starting from the time of screening using the complete blood count (CBC). The presumptive diagnosis must be made using at least two different methods of hemoglobin analysis. The hemoglobin analyses used in our center were capillary hemoglobin electrophoresis and high-performance liquid chromatography. Some mutations might coincide during the screening test, resulting in an incorrect diagnosis, for example, in Hb Malay (Codon 19 (A>G)), in CAP + 1 (A>C), or in complex genotyping, which could alter the hematological parameters, such as in the mild β-thalassemia/δ-thalassemia with normal HbA2.

There are also a few supplementary methods available to support the diagnosis. Examples include HbH inclusions, the osmotic fragility test (OF), solubility or sickling tests for HbS, and the DCIP (2.6 dichlorophenolindophenol) test for HbE [22]. The confirmation is at the DNA level either by GAP-PCR for identifying DNA deletions or gene rearrangements, direct sequencing analysis, and multiplex ligation-dependent probe amplification (MLPA). The sequential workflow might be time-consuming and costly and miss some rare causative variants, resulting in delayed genetic counseling and unresolved cases. Therefore, NGS may increase the speed of establishing a correct diagnosis and reduce costs for many genetic diseases [23,24,25].

Traditional methods using the RBC indices and Hb analysis, either by HPLC or CE, as primary screenings for thalassemia are limited because some silent thalassemia carriers with normal or borderline red cell indices/HbA2 levels may not be detected or can be missed, even in patients with combined carriers of α- and β-thalassemia. Other than false negative results, the NGS also makes a fast diagnosis by reducing the need for further referrals and repeated blood sampling. Due to financial limitations, the DNA analysis is chosen based on a differential diagnosis analysis of the patient’s phenotypic and hematological characteristics. However, our conventional methods only offer limited mutational analysis. As the phenotype varies and limits the traditional DNA analysis test, patients may sometimes miss the diagnosis. Large deletions are typically found by gap-PCR or MLPA, while point mutations and indels are typically detected by MARMS-PCR or sequencing. A homozygous β-thalassemia detected using MARMS-PCR and sequencing may not be a true homozygote but rather a compound heterozygote with a deletional β-thalassemia or a δβ-thalassemia that must be ruled out using gap-PCR, MLPA, or cascade screening [26]. Therefore, a few countries have recently introduced screening for thalassemia or hemoglobinopathies using next-generation sequencing (NGS). For example, for the Dai ethnic group from Yunnan, China, they widely use NGS to screen more than 300 α-hemoglobin and β-hemoglobin mutations, especially for identifying novel mutations and for non-invasive prenatal diagnosis. Out of the screening population, 49.5% identified as thalassemia mutation carriers [27].

NGS among thalassemia carriers is widely used, especially in China, as it helps identify unknown mutations and detect missed thalassemia simultaneously. Following the review by Suhaimi SA et al., a few studies used NGS as part of thalassemia screening [28]. For example, Shang et al. and He et al. conducted a survey on population and premarital screening programs and found that 12.1% of the variants were missed when using Hb analysis, an additional 35 couples were at risk, and 27.5% missed the carrier, respectively, based on NGS screening methods [27,29]. Similar to a study by Zhang et al. in 2019 on population screening, they identified five different novel mutations through the NGS approach, and around 2.8% of carriers were missed when using the routine method [30].

To date, more than 350 different alleles have been discovered for beta-thalassemia mutations, and over 100 different mutations have been discovered for alpha-thalassemia [31,32]. As such, molecular techniques have long been lauded as the gold standard in the diagnosis of thalassemia. However, with globalization and an increase in migration between countries near and far, the local genomic landscapes of thalassemic mutations is rapidly evolving. The intermingling and marriages of different genetic backgrounds leads to the introduction of different thalassemic mutations, leading to different patterns of coinheritance. Conventional PCR methods using targeted primers do not account for the less common or unknown mutations of any given population. NGS, on the other hand, is able to identify unknown, and even novel, mutations that are not picked up by conventional PCR methods [28]. For that reason, NGS has become increasingly popular for finding the definitive diagnosis and even for the screening of carriers of thalassemia.

NGS provides a more comprehensive and complete analysis of a patient’s genetic makeup, as it has the ability to detect multiple mutations on a single gene in a single test, whereas PCR typically only detects specific mutations that are targeted by the primer set used in the reaction. In Malaysia, at least five reference centers perform genetic testing for thalassemia, which includes GAP-PCR for alpha-thalassemia and MARMS-PCR for β-thalassemia. The MARMS-PCR method is limited to twenty different common mutations among the Malaysian population, followed by zygosity PCR analysis for positive results. Furthermore, some centers in Malaysia, for example in Hospital Kuala Lumpur, Malaysia, perform PCR for non-deletional alpha-PCR and Sanger sequencing. Thus, this requires repeated blood sampling and further referral tests, especially from regional hospitals. NGS also allows for the simultaneous detection of variants or mutations in both alpha and beta genes.

We highlighted the importance of including globin genes in the NGS analysis in most of the presented cases. From all fourteen cases, NGS was able to pick up an additional -50 G>A (HBB:c.-100G>A) that were not identified by the MARMS method, including HBA2 mutations, namely CD 79 (HBA2:c.239C>G). Other than that, there was also CD 142 (HBA2:c.427T>C), as well as other non-deletional alpha-thalassemia and alpha triplications that were not picked up by GAP-PCR methods that exclusively look for deletional alpha mutations common in our population. CD 26 (G>A) was not carried out based on the MARMS method in our cases because we routinely did not proceed with the molecular diagnostic, especially for those with an A2 in between 25 to 35%. All the additional mutations identified were clinically pathogenic and might cause further harm, especially for couples at risk. This clearly demonstrates the advantage of using NGS in the comprehensive diagnosis of thalassemia.

In addition, hemoglobin [Hb] J-Singapore was overlooked in Case 10 in the HPLC and gel electrophoresis methods. CD 79 (HBA2:c.239C>G) was discovered when NGS was used. Hb J-Singapore is a rare α-globin chain variant that has been reported in Singapore, Malaysia, and Thailand [33]. No extensive study was performed on the prevalence of this Hb variant. However, a few cases were reported among Malaysian family members living in Singapore, one of them a Malaysian and Thai woman [33,34,35]. In our case, it showed an abnormal band (presence of a fast band in the Hb Bart region with a prominent A2/E band) during alkaline gel electrophoresis, while there was no abnormal Hb peak at a retention time of 1.50–1.90, based on high-performance liquid chromatography [HPLC]. Furthermore, no capillary electrophoresis was performed on this patient. The variant eluted in the P3 window might have been overlooked during routine Hb analysis [35]. This case was an example of how DNA sequencing data complements identifying Hb variants, particularly for those that undergo posttranslational modifications. This Hb J-Singapore may be clinically relevant when it co-inherits with the alpha-thalassaemia-1 or other α-globin gene variants [33]. In this case, the heterogeneity of CD 41/42 may produce a thalassemia major phenotype. However, as it has co-inheritance with Hb J-Singapore, which acts as a secondary modifier that ameliorates the imbalance of the globin chain, it helps in the production of the intermedia phenotypic features.

The primary and significant role of NGS is particularly important in order to make a conclusive diagnosis of thalassemia and evaluate the unresolved cases. As seen in case 12, the patient had moderate anemia on her post-transfusion sample, which did not correspond with the results of her MARMS, which found a beta-thalassemia trait phenotype. Patients with -50 (G>A) carriers had normal hematological parameters, while the compound heterozygotes for -50 and β-thalassemia had a similar hematological presentation as that of the β-thalassemia trait [36]. By identifying that the patient had additional triplicated α-globin genes, which played a vital modifier role in exacerbating her phenotypic β-thalassemia by affecting the erythroid maturation and causing an imbalance between the α- and β-globin chains, a thalassemia intermedia phenotype was produced [37,38]. This was similar to the case reported by Steinberg-Shemer, O. et al., where whole exome sequencing (WES) was described as a useful tool in unraveling patients’ diagnoses, especially with atypical presentation, and additional secondary modifiers that might exacerbate the disease presentation were determined [39]. Therefore, extensive genetic sequencing on her sample elucidated a precise genetic diagnosis. However, standard multiplex gap-PCR and MARMS-PCR were unable to detect this alpha triplication. Thus, further single-tube multiplex PCR for alpha triplication should be conducted. This is especially important to render a conclusive diagnosis for the patient and provide correct clinical management. The diagnostic NGS-based method made the detection of rare genetic variants of α- and β-globin genes available to us.

In case 13, Hb Malay was initially described in 1989 as originating from Malaysia and was found in around 15% of the Malaysian population [40,41]. However, as Hb Malay co-eluted within HbA in CE and HPLC, molecular analysis can only make a definitive diagnosis. Patients with Hb Malay usually have a mild b-thalassemia phenotype, with mild microcytosis and elevated HbA2 levels [42,43].

In the genomic era, NGS may play a significant role in screening and diagnosing thalassemia, especially by filling the gaps and solving complex genomics riddles. However, there are still a few challenges in implementing NGS in thalassemia, especially in a developing country with limited funding resources. Nevertheless, NGS has improved precision and covers an extensive spectrum of mutations. The most common NGS technologies used worldwide consist of whole exome sequencing, which covers the entire exome, whole genome sequencing, which covers all genes and non-coding DNA, and targeted region gene sequencing, which covers around 10–500 genes [44,45].

Few findings in the cases mentioned above demonstrate the need for multiple molecular methods to confirm the diagnosis, especially when identifying alpha- and beta-thalassemia. Even for alpha-thalassemia, two methods are required, as the GAP-PCR method is unable to detect those with non-deletional mutations. NGS, using amplicons, has proven to be an efficient tool that can simultaneously detect α- and β-thalassemia variants and resolve complicated cases of thalassemia that would have stayed undiagnosed [46,47]. This study used Illumina NGS based on the sequencing of the synthesis and fluorescence excitation method. Therefore, the optical instrument was used to record the fluorescence signal, convert it into bases, and align it to a reference genome with the bits of help from bioinformatics to analyze the possibility of indels, the significant copy number variations, and the single nucleotide polymorphism [48].

**Figure 4:**
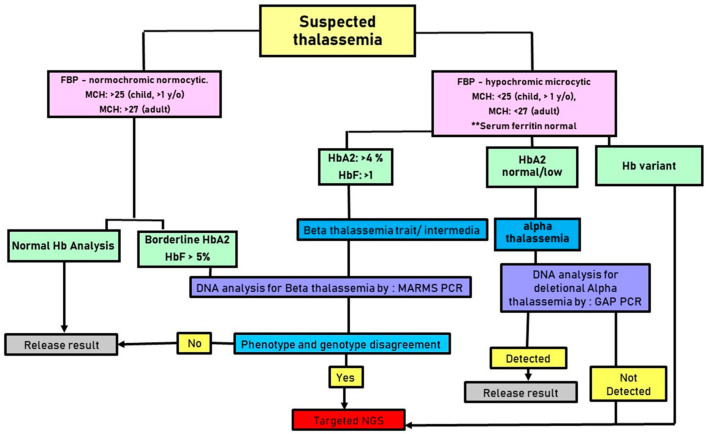
The proposed workflow for the screening and diagnosis of thalassemia that incorporates the NGS method in a developing country.

A study by Shang et al. also described targeted NGS as the most cost-effective treatment for thalassemia because of the modest cluster sizes. For uncommon copy number variation (CNV) identification and breakpoint estimate, a targeted NGS should ideally be able to detect point mutations in HBA, HBB, HBD, and HBG. It should also include uniform reads spanning the neighboring genes. Direct detection of common CNV is accurate when CNV spanning reads are added [29]. Thus, NGS is an efficient technology that facilitates thalassemia screening. The diagnosis of α-thalassemia is difficult, since the α-globin gene is highly variable. Hence, the choice of the diagnostic approach depends on the laboratory’s equipment, competence, associated costs, and facilities [49]. Figure 4 illustrates on the proposed workflow involving targeted NGS, especially in a low-income country.

Overall, while NGS had the ability to detect a wider range of mutations, several pitfalls were identified. One of the main limitations was that our target part only covered three globin genes, which were *HBA1*, *HBA2*, and *HBB*; therefore, the panel design still did not include all potentially significant genes that might influence the patients’ phenotypes. Aside from that, it had the ability to only detect a few specific globin genes involving the primary modifier and secondary modifier but none for the tertiary modifier. Other than being more expensive and requiring more DNA samples to perform, compared to conventional methods, large HBA gene deletions were harder to detect using NGS methods, due to their breakpoints being embedded within long, repetitive sequences [50]. There was still a potential for errors involving the quality of the DNA samples, sequencing, and the bioinformatics pipeline.

Therefore, additional whole exon or whole genome sequencing might still be required, especially for those with discordant phenotypes and genotypes. Otherwise, the aim of this study was initially to illustrate the practicality and precise method to detect known variants related to clinical diagnosis, especially in a developing country.

## 5. Conclusions

In conclusion, to our knowledge, the results of this study should be heeded, as this is the first report on the usefulness of targeted NGS concerning the biological and phenotypic features of thalassemia, especially in a developing population, though it only involves a small cohort of the population. Therefore, a comprehensive targeted next-generation sequencing (NGS) test would immensely facilitate the diagnosis of thalassemia.

## Figures and Tables

**Table 1:** Incidence of alpha- and beta-thalassemia across several developing countries.

Alpha-Thalassemia			
Countries	Genotype Variants	Incidence	References
Malaysia			
Malay	αα/-^SEA^	44.3%	Azma et al. [9]
	αα/-α^3.7^	33.5%	
	-α^3.7^/-^SEA^	7.1%	
	αα/αα^CS^	6.2%	
Chinese	αα/-^SEA^	83.4%	
	αα/-α^3.7^	4.9%	
	-α^3.7^/-^SEA^	7.2%	
	αα/αα^CS^	0.5%	
	-^SEA^/-^SEA^	2.0%	
Indian	αα/-^SEA^	5%	
	αα/-α^3.7^	90%	
Kadazandusun	-α^3.7^*/*αα	33.6% (42/125)	Tan et al. [10]
Thailand	αα/-α^3.7^	17.7%	Lithanatudom et al. [11]
	αα/-^SEA^	3.5%	
	_α^CS^/αα	2.1%	
China	-^SEA^/αα	31.53%	Wang et al. [12]
	-α^4.2^/αα	11.15%	
	-α^3.7^/αα	11.02%	
	-α^WS^/αα	5.48%	
Indonesia	SEA	16.2%	Setianingsih et al. [13]
	-α^3.7^	14.1%	
	Cd 59 (GGC>GAC)	3.0%	
	Cd 22 (GGC>GGT)	1.0%	
	Hb Constant Spring	1.0%	
Beta-thalassemia			
Countries	Genotype variants	Incidence	References
Malaysia			
Malay	IVS I-5	15.3%	George et al. [14]
	CD 26	32.1%	
	IVS I-1	8.0%	
	CD 19	7.3%	
	CD 41/42	3.6%	
	CD 17	1.5%	
	-29	1.5%	
	CD 8/9	0.7	
	CAP + 1	0%	
	Homozygous CD 19/CD 19	0.7%	
	Homozygous CD 71/72	0.7%	
	IVS I-1/CD 19	0.7%	
	CD 26/CD 26	3.6%	
	CD 26/IVS I-5	9.5%	
	CD 26/IVS I-1	5.1%	
	CD 26/CD 19	3.6%	
Kadazandusun	45 kb Filipino β-deletion	12.8% (16/125)	Tan et al. [10]
Thailand	Codon 41/42 (-TCTT)	37.5%	Traivaree et al. [15]
	Codon 17 (A>T)	26.1%	
	IVS-I-5 (G>C)	8.0%	
	IVS-II-654 (C>T)	6.8%	
	IVS-I-I (G>T)	4.5%	
	Codon 71/72 (+A)	2.3%	
	Codon 35 (C > A)	4.5%	
	Initiation codon mutation (c.2T > G)	1.1%	
	Codon 15 (G>A)	1.1%	
	Codon 19 (A>G)	1.1%	
	Codon 27/28 (+C)	1.1%	
	Codon 123/124/125	1.1%	
	(-ACCCCACC)		
	3.2 kb deletion	4.5%	
China	CD41/42	30.27%	Wang et al. [12]
	-28	2.56%	
	IVS-II-654	1.02%	
Indonesia	IVS-I-5 (G>C)	43.5%	Rujito et al. [16]
	Codon 26 (G>A)	28.2%	
	IVS-I-1 (G>A)	5.0%	
	Codon 15 (TGG>TAG)	3.8%	
	IVS-I-1(G>T)	3.1%	
	Codon 35 (-C)	2.4%	
	Codon 41/42 (-TTCT)	1.0%	
	Codon 8/9 (+G)	1.0%	
	Codon 19 (AAC > AGC)	0.7%	
	Codon123/124/125	0.5%	
	(-ACCCCACC)		

**Table 2:** List of patients, FBC results, HB analysis results, molecular status, and DNA/RNA sequencing data.

Cases		FBC				Hb Analysis (HPLC)			Molecular		Devyser NGS
		RBC	Hb (g/L)	MCV (80-100 fl)	MCH (pg/Cell) <25, Child <27, Adult	HbA	HbA2	HbF	MARMS (Beta-Thalassemia)	GAP-PCR (Alpha-Thalassemia)	
1	3-year-old female	3.06	6.5	66.0	21.2	5.9	46.1	48	CD 26 (G>A) IVS I-1 (G>T)	Not performed	CD 26 (HBB:c.79G>A) IVS I-1 (HBB:c.92 + 1G>T)
2	27-year-old female	3.98	8.6	66.1	21.6	51.6	43.6	4.8	CD 26 (G>A) Poly A (A>G)	Not performed	CD 26 (HBB:c.79G>A) Poly A (HBB:c.*112A>G)
3	3-year-old female	5.00	12.5	72.4	25	71.7	24.7	3.6	Not performed	aa/a4.2	aa/a4.2 CD 26 (HBB:c.79G>A)
4	4-year-old male	5.59	9.7	55.5	17.4	95.9	2.9	1.2	Not performed	SEA deletion	SEA deletion
5	23-year-old male	6.02	13.4	69.6	22.3	97.1	2.6	0.3	Not performed	THAI deletion	THAI deletion
6	36-year-old female	6.06	14.2	76.2	23.4	96	2.8	1.2	Not performed	aa/a3.7	aa/a3.7
7	27-year-old female	4.98	7.5	55	15.1	81.9	14.6	3.5	CD 26 (*HBB*:c.79G>A)	SEA deletion	SEA deletion CD 26 (HBB:c.79G>A) CD 142 (HBA2:c.427T>C)
						Hb Bart’s disease. HbE trait with Hb Constant Spring					
8	29-year-old female	5.23	10.7	65	20.5	92.1	5.5	2.4	Heterozygous IVS I-5(G>C)	Not performed	IVS I-5 (HBB:c.92 + 5G>C)
9	30-year-old female	3.37	8.0	73	23.7	Not available (Refer by other hospital)			Homozygous -28 (A>G)	Not performed	-28 (A>G) (HBB:c.-78A>G)
10	18-year-old female	5.72	8.8	53.5	15.4	90.3	4.3	5.4	Heterozygous CD 41/42	Not performed	CD 41/42; (HBB:c.126_129del) CD 79 (HBA2:c.239C>G)
						Abnormal band (Presence of fast band at Hb Bart’s region with prominent A2/E band) on Alkaline gel electrophoresis					
11	7-year-old female	5.53	10.8	62	19.5	55.2	42.6	2.2	CD 26 (G>A) CAP + 1 (A>C)	Not performed	CD 26 (HBB:c.79G>A) CAP + 1 (HBB:c.-50A>C); CD 142 (HBA2:c.427T>C)
12	35-year-old female	3.3	6.8	66.4	20.6	92.1	5.8	2.1	Heterozygous CD 8/9 (+G)	Not performed	CD 8/9 (HBB:c.27dup); -50 G>A (HBB:c.-100G>A) 4.2 triplication
						Recently transfuse sample					
13	8-year-old male	5.93	11.2	62.1	18.9	93.9	4.7	1.4	Heterozygous CD19 (A>G)	Not performed	CD19 (HBB:c.59A>G)
14	5-month-old male	1.87	4.6	71.1	24.6	1.9	2.4	95.7	CD17 (A>T) IVS II-654 (C>T)	Not performed	CD17 (HBB:c.52A>T) IVS II-654 (HBB:c.316-197C>T)

* Hb, hemoglobin; HbA, hemoglobin alpha; HbA2, hemoglobin alpha 2; MCV, mean cell volume; MCH, mean cell hemoglobin; MARMS, multiplex amplification refractory mutation system; PCR, polymerase chain reaction.

## Data Availability

The data presented in this study are available upon request from the corresponding author.

## References

[1] Weatherall D.J. (2004). The Thalassemias: The Role of Molecular Genetics in an Evolving Global Health Problem. Am. J. Hum. Genet..

[2] Cao A., Kan Y.W. (2013). The Prevention of Thalassemia. Cold Spring Harb. Perspect. Med..

[3] Ngim C.F., Ibrahim H., Lai N.M., Ng C.S. (2015). A single centre study on birth of children with transfusion-dependent thalassaemia in Malaysia and reasons for ineffective prevention. Prenat. Diagn..

[4] Ibrahim M. (2019). Malaysian thalassaemia registry report 2018. Minist. Health Malays..

[5] Ismail A., Campbell M.J., Ibrahim H.M., Jones G.L. (2006). Health Related Quality of Life in Malaysian children with thalassaemia. Health Qual. Life Outcomes.

[6] Kwiatkowski J.L., Kim H.-Y., Thompson A.A., Quinn C.T., Mueller B.U., Odame I., Giardina P.J., Vichinsky E.P., Boudreaux J.M., Cohen A.R. (2012). Chelation use and iron burden in North American and British thalassemia patients: A report from the Thalassemia Longitudinal Cohort. Blood.

[7] Lee J.S., Cho S.I., Park S.S., Seong M.W. (2021). Molecular basis and diagnosis of thalassemia. Blood Res..

[8] Elizabeth G., Ann T.J.A.M. (2010). Genotype-Phenotype Diversity of Beta-Thalassemia in Malaysia: Treatment Options and Emerging Therapies. Med. J. Malays..

[9] Azma R.Z., Ainoon O., Hafiza A., Azlin I., Farisah A.R.N., Hidayati S.N., Hamidah H.N. (2014). Molecular characteristic of alpha thalassaemia among patients diagnosed in UKM Medical Centre. Malays. J. Pathol..

[10] Tan J.-A.M.A., Lee P.-C., Wee Y.-C., Tan K.-L., Mahali N.F., George E., Chua K.-H. (2010). High prevalence of alpha- and beta-thalassemia in the kadazandusuns in east Malaysia: Challenges in providing effective health care for an indigenous group. J. Biomed. Biotechnol..

[11] Lithanatudom P., Khampan P., Smith D.R., Svasti S., Fucharoen S., Kangwanpong D., Kampuansai J. (2016). The prevalence of alpha-thalassemia amongst Tai and Mon-Khmer ethnic groups residing in northern Thailand: A population-based study. Hematology.

[12] Wang Z., Sun W., Chen H., Zhang Y., Wang F., Chen H., Zhou Y., Huang Y., Zhou X., Li Q. (2021). Prevalence and molecular spectrum of α- and β-globin gene mutations in Hainan, China. Int. J. Hematol..

[13] Setianingsih I., Harahap A., Nainggolan I.M. (2003). Alpha thalassaemia in Indonesia: Phenotypes and molecular defects. Adv. Exp. Med. Biol..

[14] George E., Teh L.K., Rosli R., Lai M.I., Tan J. (2012). Beta Thalassaemia Mutations in Malays: A Simplified Cost-effective Strategy to Identify the Mutations. Malays. J. Med. Health Sci..

[15] Boonyawat B., Monsereenusorn C., Traivaree C. (2014). Molecular analysis of beta-globin gene mutations among Thai beta-thalassemia children: Results from a single center study. Appl. Clin. Genet..

[16] Rujito L., Basalamah M., Mulatsih S., Sofro A.S.M. (2015). Molecular Scanning of β-Thalassemia in the Southern Region of Central Java, Indonesia; a Step Towards a Local Prevention Program. Hemoglobin.

[17] Leckngam P., Limweeraprajak E., Kiewkarnkha T., Tatu T. (2017). The Hb E (HBB: C.79G>A), Mean Corpuscular Volume, Mean Corpuscular Hemoglobin Cutoff Points in Double Heterozygous Hb E/– –SEA α-Thalassemia-1 Carriers are Dependent on Hemoglobin Levels. Hemoglobin.

[18] Yokoyama A., Nakamaki T., Yamada K., Koike M., Tomoyasu S., Hirayama N., Tsuruoka N., Harano T. (1993). Beta 0-thalassemia trait (IVS-I-1 G-->T) in a Japanese family. Intern. Med..

[19] Wisedpanichkij R., Jindadamrongwech S., Butthep P. (2015). Identification of Hb Constant Spring (HBA2: C.427T > C) by an Automated High Performance Liquid Chromatography Method. Hemoglobin.

[20] Alwi Z.B., Syed-Hassan S.N.R.K. (2022). Thalassemia in Malaysia. Hemoglobin.

[21] Murad H., Moassas F., Fakseh N.A.L. (2021). A rare gene variation cap +1 (A>C) (HBB: c. -50A>C) associated with codon 5 (-CT) (HBB: c.17_18delCT) mutation in Syrian family. Mol. Genet. Genom. Med..

[22] Fauzi M., Yusoff M., Rahayu E., Tohit M., Hashim H., Seman Z. (2021). Evaluation of Dichlorophenolindophenol (DCIP) Test for Haemoglobin E (Hb E) in Normal Red Cell Indices Individuals. Malays. J. Med. Health Sci..

[23] Farmakis D., Porter J., Taher A., Cappellini M.D., Angastiniotis M., Eleftheriou A. (2022). 2021 Thalassaemia International Federation Guidelines for the Management of Transfusion-dependent Thalassemia. HemaSphere.

[24] Wong S.L., Ng H.P., Tam P.Y., Hung L.C., Muda Z. (2003). Report Management of Thalassaemia. Health Technology Assessment Unit Medical Development Division Ministry of Health Malaysia. https://www.moh.gov.my/moh/resources/autodownloadimages/587f136ce4807.pdf.

[25] Traeger-Synodinos J., Harteveld C.L., Old J.M., Petrou M., Galanello R., Giordano P., Angastioniotis M., De la Salle B., Henderson S., May A. (2015). EMQN Best Practice Guidelines for molecular and haematology methods for carrier identification and prenatal diagnosis of the haemoglobinopathies. Eur. J. Hum. Genet..

[26] Hassan S., Bahar R., Johan M.F., Hashim E.K.M., Abdullah W.Z., Esa E., Hamid F.S.A., Zulkafli Z. (2023). Next-Generation Sequencing (NGS) and Third-Generation Sequencing (TGS) for the Diagnosis of Thalassemia. Diagnostics.

[27] He J., Song W., Yang J., Lu S., Yuan Y., Guo J., Zhang J., Ye K., Yang F., Long F. (2017). Next-generation sequencing improves thalassemia carrier screening among premarital adults in a high prevalence population: The Dai nationality, China. Genet. Med..

[28] SSuhaimi A., Zulkipli I.N., Ghani H., Abdul-Hamid M.R.W. (2022). Applications of next generation sequencing in the screening and diagnosis of thalassemia: A mini-review. Front. Pediatr..

[29] Shang X., Peng Z., Ye Y., Asan, Zhang X., Chen Y., Zhu B., Cai W., Chen S., Cai R. (2017). Rapid Targeted Next-Generation Sequencing Platform for Molecular Screening and Clinical Genotyping in Subjects with Hemoglobinopathies. EBioMedicine.

[30] Zhang H., Li C., Li J., Hou S., Chen D., Yan H., Chen S., Liu S., Yin Z., Yang X. (2019). Next-generation sequencing improves molecular epidemiological characterization of thalassemia in Chenzhou Region, P.R. China. J. Clin. Lab. Anal..

[31] Kwaifa I.K., Lai M.I., Noor S.M. (2020). Non-deletional alpha thalassaemia: A review. Orphanet J. Rare Dis..

[32] Jaing T.H., Chang T.Y., Chen S.H., Lin C.W., Wen Y.C., Chiu C.C. (2021). Molecular genetics of β-thalassemia: A narrative review. Medicine.

[33] Panyasai S., Permsripong N., Pornprasert S. (2018). Hemoglobin J-Singapore [α79(EF8)Ala→Gly, GCG>GGG] in a pregnant Thai woman. J. Med. Assoc. Thail..

[34] Blackwell R.Q., Boon W.H., Liu C.-S., Weng M.-I. (1972). Hemoglobin J Singapore: α78 Asn → Asp; α79 Ala → Gly. Biochim. Biophys. Acta Protein Struct..

[35] O’Brien D.A., Clark B., Rai D.K. (2007). Alpha78(EF7)Asn-->Asp is a posttranslational modification in Hb J-Singapore [alpha78(EF7)Asn-->Asp;alpha79(EF8)Ala-->Gly]. Hemoglobin.

[36] Zhao Y., Jiang F., Li D.Z. (2020). Hematological Characteristics of β-Globin Gene Mutation -50 (G>A) (HBB: c.-100G>A) Carriers in Mainland China. Hemoglobin.

[37] Luo X., Zhang X.M., Wu L.S., Chen J., Chen Y. (2021). Prevalence and clinical phenotype of the triplicated α-globin genes and its ethnic and geographical distribution in Guizhou of China. BMC Med. Genom..

[38] Rameli N., Ramli M., Zulkafli Z., Hassan M.N., Yusoff S.M., Noor N.H.M., Hussin S., Kamarudin N.K.M., Yusoff Y.M., Bahar R. (2022). Challenges in diagnosis of Beta Thalassemia syndrome: The Importance of Molecular Diagnosis. Oman Med. J..

[39] Steinberg-Shemer O., Ulirsch J., Noy-Lotan S., Krasnov T., Attias D., Dgany O., Laor R., Sankaran V.G., Tamary H. (2017). Whole-exome sequencing identifies an α-globin cluster triplication resulting in increased clinical severity of β-thalassemia. Cold Spring Harb. Mol. Case Stud..

[40] Yang K.G., Kutlar F., George E., Wilson J.B., Kutlar A., Stoming T.A., Redondo J.M.G., Huisman T.H.J. (1989). Molecular characterization of β-globin gene mutations in Malay patients with Hb E-β-thalassaemia and thalassaemia major. Br. J. Haematol..

[41] Ma S.K., Chow E.Y., Chan A.Y., Kung N.N., Waye J.S., Chan L.C., Chui D.H. (2000). Thalassemia Intermedia Caused by Compound Heterozygosity for Hb Malay (Codon 19 AAC→AGC; Asn→Ser) and Codons 41/42 (-CTTT) 0-Thalassemia Mutation. J. Hematol..

[42] Shahida N.S., Nazri M.H., Haslina N.M., Zaidah W.A. (2017). The Diagnosis of Beta Thalassemia with Borderline HbA2 Level among Kelantan Population. J. Blood Disord. Transfus..

[43] Amran H.S., Sarijan N., Sathar J.S., Noor S.M. (2018). Case Series of Homozygous and Compound Heterozygosity of Hb Malay, the Diagnostic Features and Transfusion Requirements. J. Biomed. Clin. Sci..

[44] Pervez M.T., Hasnain M.J.U., Abbas S.H., Moustafa M.F., Aslam N., Shah S.S.M. (2022). A Comprehensive Review of Performance of Next-Generation Sequencing Platforms. BioMed Res. Int..

[45] Behjati S., Tarpey P.S. (2013). What is next generation sequencing?. Arch. Dis. Child. Educ. Pract. Ed..

[46] Sabath D.E. (2017). Molecular Diagnosis of Thalassemias and HemoglobinopathiesAn ACLPS Critical Review. Am. J. Clin. Pathol..

[47] Achour A., Koopmann T.T., Baas F., Harteveld C.L. (2021). The Evolving Role of Next-Generation Sequencing in Screening and Diagnosis of Hemoglobinopathies. Front. Physiol..

[48] Synthesis (SBS) Technology. Sequencing Technology|Sequencing by Synthesis. https://sapac.illumina.com/science/technology/next-generation-sequencing/sequencing-technology.html.

[49] Vijian D., Rahman W.S.W.A., Ponnuraj K.T., Zulkafli Z., Noor N.H.M. (2021). Molecular Detection of Alpha Thalassemia: A Review of Prevalent Techniques. Medeni. Med. J..

[50] Clark B.E., Shooter C., Smith F., Brawand D., Thein S.L. (2017). Next-generation sequencing as a tool for breakpoint analysis in rearrangements of the globin gene clusters. Int. J. Lab. Hematol..

